# Invertase-producing bacteria and the sweetness content dataset of Cilembu sweet potatoes (Ipomoea batatas (L.) Lam.) grown in various agroecosystems

**DOI:** 10.1016/j.dib.2024.110086

**Published:** 2024-01-23

**Authors:** Eso Solihin, Syaiful Anwar, Dwi Andreas Santosa, Budi Nugroho, Purwono, Rija Sudirja, Haris Maulana, Nadia Nuraniya Kamaluddin, Agung Karuniawan

**Affiliations:** a Department of Soil Science and Land Resource, Faculty of Agriculture, IPB University, Dramaga, Bogor 16680, Indonesia; b Departement of Soil Science and Land Resource, Faculty of Agriculture, Universitas Padjadjaran, Jatinangor, Sumedang 45363, Indonesia; c Department of Agronomy and Horticulture, Faculty of Agriculture, IPB University, Dramaga, Bogor 16680, Indonesia; d Research Center for Horticultural and Estate Crops, Research Organization for Agriculture and Food, National Research and Innovation Agency, Cibinong, Bogor 16911, West Java, Indonesia; e Department of Agronomy, Faculty of Agriculture, Universitas Padjadjaran, Jatinangor, Sumedang 45363, Indonesia

**Keywords:** Sweet potato, West Java, Yield, Yield quality

## Abstract

Cilembu sweet potato is one of Indonesia's leading agricultural commodities. The high carbohydrate content in sweet potatoes has the potential to change into sugar (glucose, sucrose, and fructose) during storage. The level of sweetness is one of the characteristics that determines the quality of sweet potatoes. The sweetness level of sweet potatoes is influenced by various factors, including genetics, environment, and their interactions. Apart from that, the role of invertase-producing bacteria in breaking down carbohydrates into sugars is very important. Information regarding the number of invertase-producing bacteria in Cilembu sweet potatoes and their activity during storage is still limited. This research aimed to determine the number and activity of invertase-producing bacteria in Cilembu sweet potatoes and estimate the relationship between activity and total invertase-producing bacteria during storage of Cilembu sweet potatoes. The results showed significant differences between the number and activity of invertase-producing bacteria at each storage time. There is a relationship between sugar levels and invertase-producing bacteria. Sucrose levels had a negative and significant correlation with fructose levels (-0.56) and invertase-producing bacteria (-0.58). Glucose levels were significantly and positively correlated with fructose levels (0.91) and invertase-producing bacteria (0.88). Fructose levels also significantly and positively correlated with invertase-producing bacteria (0.95). This information can be used as a reference in determining the quality of sweet potatoes directly and indirectly.

Specifications Table

**Table:** 

Subject	Data Article (Agricultural and Biological Science)
Specific subject area	Agronomy and Crop Science, Soil Science, and Enzyme Activities
Data format	Analyzed data
Type of data	Table and Figure
Data collection	This data was collected by taking tuber samples from four different environments. Data was taken from each tuber storage time. The data includes the invertase-producing bacteria population, invertase activity, and sugar content. Determination of invertase producing bacteria was observed through direct observation by counting the total microbes. Observations were made by counting the number of bacterial colonies that changed color to red when sprayed with 0.1 % TTC. Invertase activity was determined quantitatively by measurement using UV–Vis spectrophotometry. The sugar levels observed were sucrose, glucose, and fructose levels which were determined by measuring the sample extract using HPLC.
Data source location	Samples were taken from four environments in West Java, Indonesia. Latitude 6054′13.1″S, longitude 107,050′41.7′'E (Sumedang - Paddy field); latitude 6054′17.2″S, longitude 107,050′39.7″E (Sumedang - Dry field); latitude 703.4440″S longitude 107,038.7470″E (Bandung - Paddy field); latitude 703.6310″S, longitude 107,038.7480″E (Bandung - Dry field). Altitude: 845 m asl (Sumedang - Paddy field); 863 m asl (Sumedang - Dry field). 920 m asl (Bandung - Paddy field); 965 m asl (Bandung - Dry field).
Data accessibility	Repository name: Mendeley Data Data identification number: 10.17632/59ng6845dd.1 Direct URL to data: https://data.mendeley.com/datasets/59ng6845dd/1

## 1 Value of the Data

• This database provides a valuable resource describing the amount of sucrose, glucose, fructose, and bacterial invertase activity in sweet potatoes at various storage times.
• The dataset in this article supplies data to researchers, farmers, and industry users on the yield and yield quality of cilembu sweet potato genotypes grown in different locations of West Java, Indonesia.
• This database can be used to identify the relationship between types of sugar (sucrose, fructose and glucose) with the total activity of invertase-producing bacteria.
• This data can be an impetus for developing sweet potato research that leads to yield quality.
• Researchers studying sweet potatoes' sweetness level can use this data.

## 2 Background

Yield and yield quality (sweetness level) are important characteristics in selecting superior varieties. In sweet potatoes, the sweetness level is one of the consumer parameters in choosing and consuming them. Yields and yield quality are greatly influenced by genetics, environment, and their interactions [1]. In other research, differences in planting environment caused differences in the yield quality, especially the sweetness level of sweet potatoes [2]. In the food industry, information regarding sweetness levels is very useful because it relates to processed products' quality. The relationship between the planting environment and the yield quality is very valuable because it is closely related to large-scale development programs [3]. Invertase is one of the key enzymes in partitioning and modulating starch and sugar components in sweet potatoes [4]. This enzyme hydrolyzes sucrose and other oligosaccharides containing β-fructose. It plays an important role in growth and development processes, detecting the presence of sugars and resistance to disease and stress [5,6]. Despite this understanding, few studies have explored the function of invertase in sweet potatoes. So, conducting a special study regarding the total activity of invertase-producing bacteria in Cilembu sweet potatoes is necessary.

## 3 Data Description

**Fig. 1:**
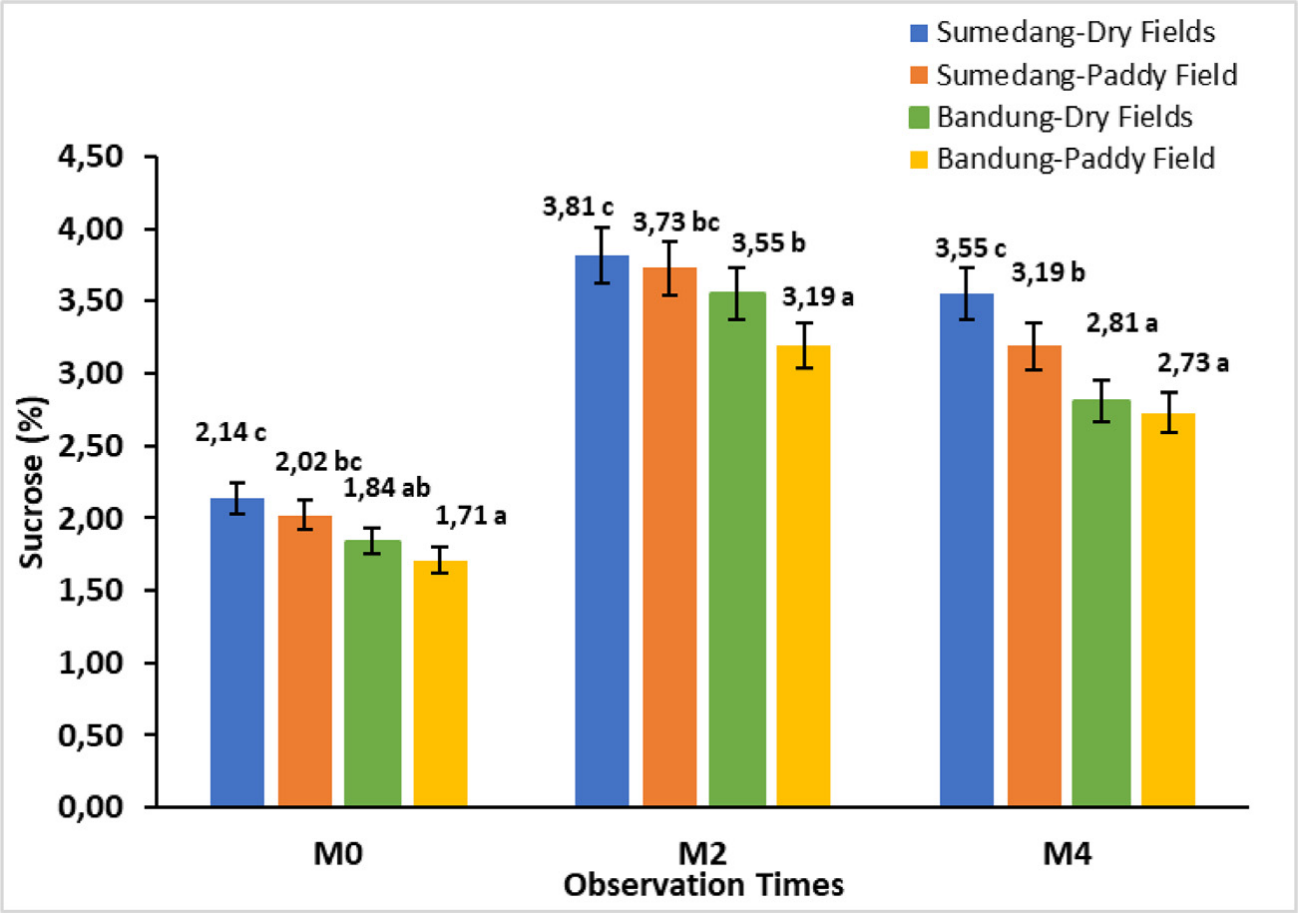
Bar chart of the storage time effect on sucrose content in Cilembu sweet potatoes at various locations. M0= 0 weeks after storage, M2= 2 weeks after storage, M4= 4 weeks after storage. Numbers in the same bar chart followed by the same letter are not significantly different based on the Duncan test at the 5 % level.

The data in Fig. 1 shows the effect of storage time on the sucrose content in Cilembu sweet potatoes in four different environments. The sucrose content of Cilembu sweet potato in each environment showed significant differences at each storage time. The sucrose content of sweet potatoes two weeks after storage (M2) showed an increase between 78.04 % to 92.93 %. In contrast, four weeks after storage (M4) decreased between 6.82 to 20.85 %.

**Fig. 2:**
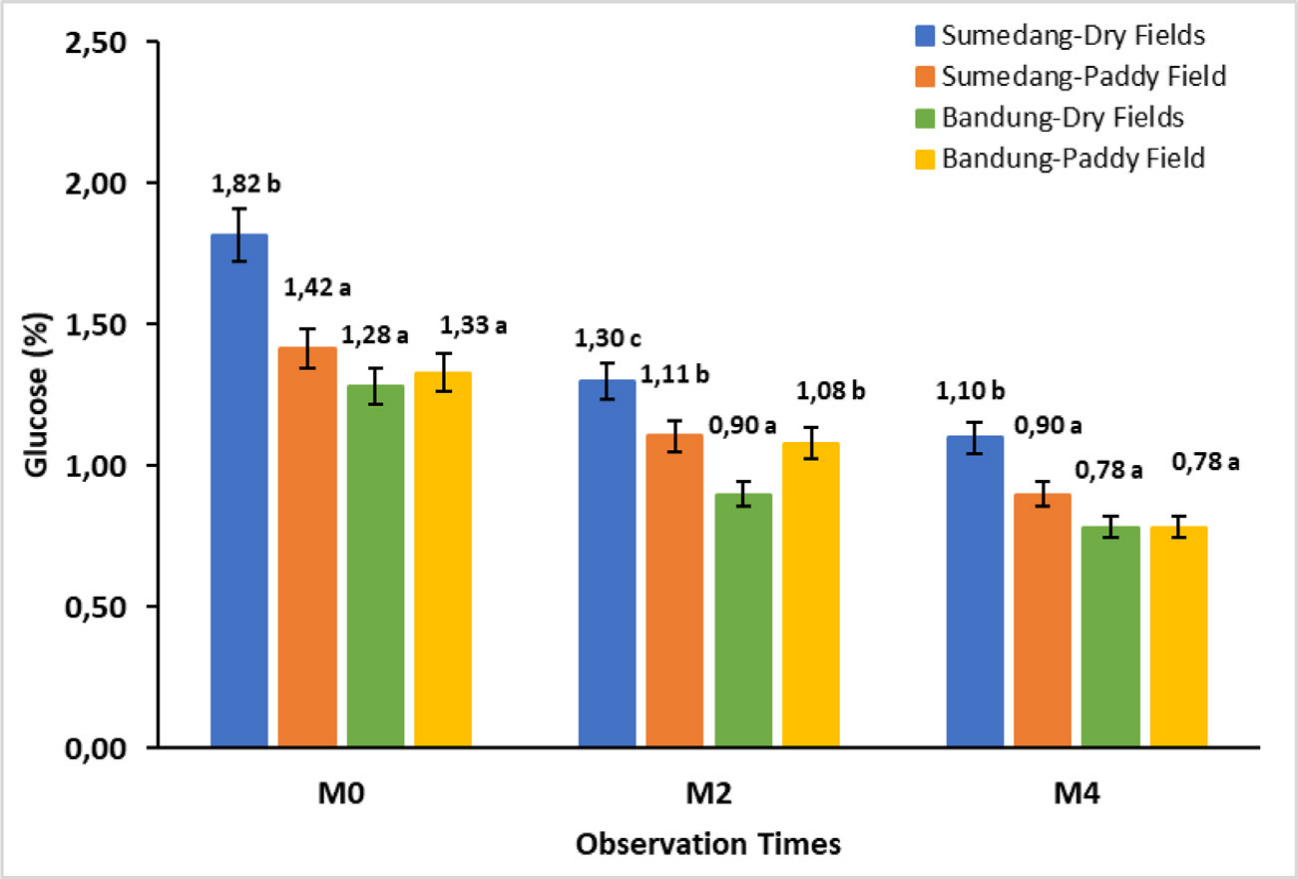
Bar chart of the storage time effect on glucose content in Cilembu sweet potatoes at various locations. M0= 0 weeks after storage, M2= 2 weeks after storage, M4= 4 weeks after storage. Numbers in the same bar chart followed by the same letter are not significantly different based on the Duncan test at the 5 % level.

The data in Fig. 2 shows the effect of storage time on the glucose content of Cilembu sweet potatoes in four different environments. The glucose content in Cilembu sweet potatoes in various environments showed significant differences at each storage time. The glucose content decreased during storage, from 18.80 % to 29.69 % in M2 and 13.33 % to 27.78 % in M4.

**Fig. 3:**
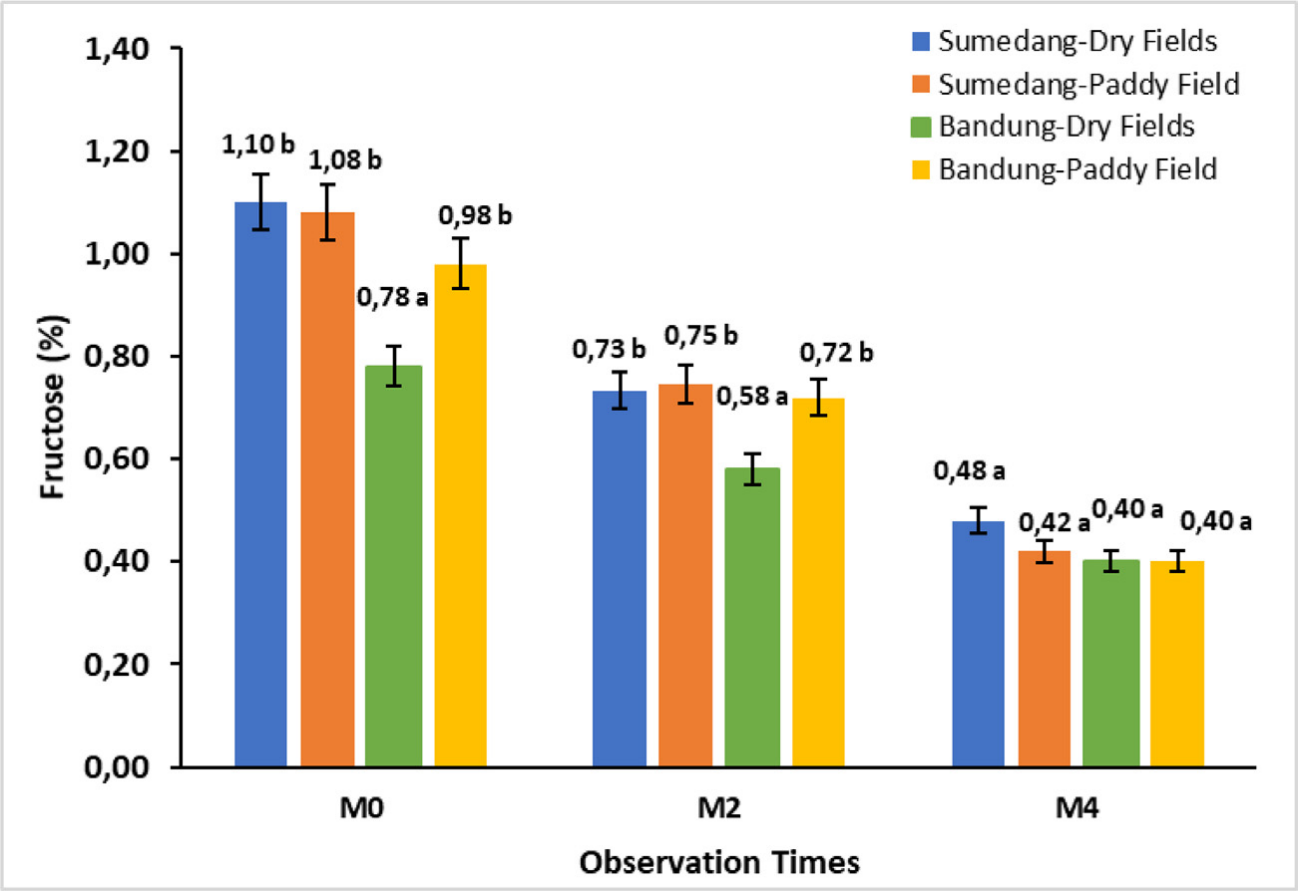
Bar chart of the storage time effect on fructose content in Cilembu sweet potatoes at various locations. M0= 0 weeks after storage, M2= 2 weeks after storage, M4= 4 weeks after storage. Numbers in the same bar chart followed by the same letter are not significantly different based on the Duncan test at the 5 % level.

The data in Fig. 3 shows the effect of storage time on the fructose content in Cilembu sweet potatoes in four different environments. The fructose content in M0 and M2 showed a significant difference, while M4 showed no significant difference. The fructose content decreased during storage, namely around 25.64 % to 33.64 % at M0, while at M4, the decrease ranged from 31.03 % to 44.44 %.

**Fig. 4:**
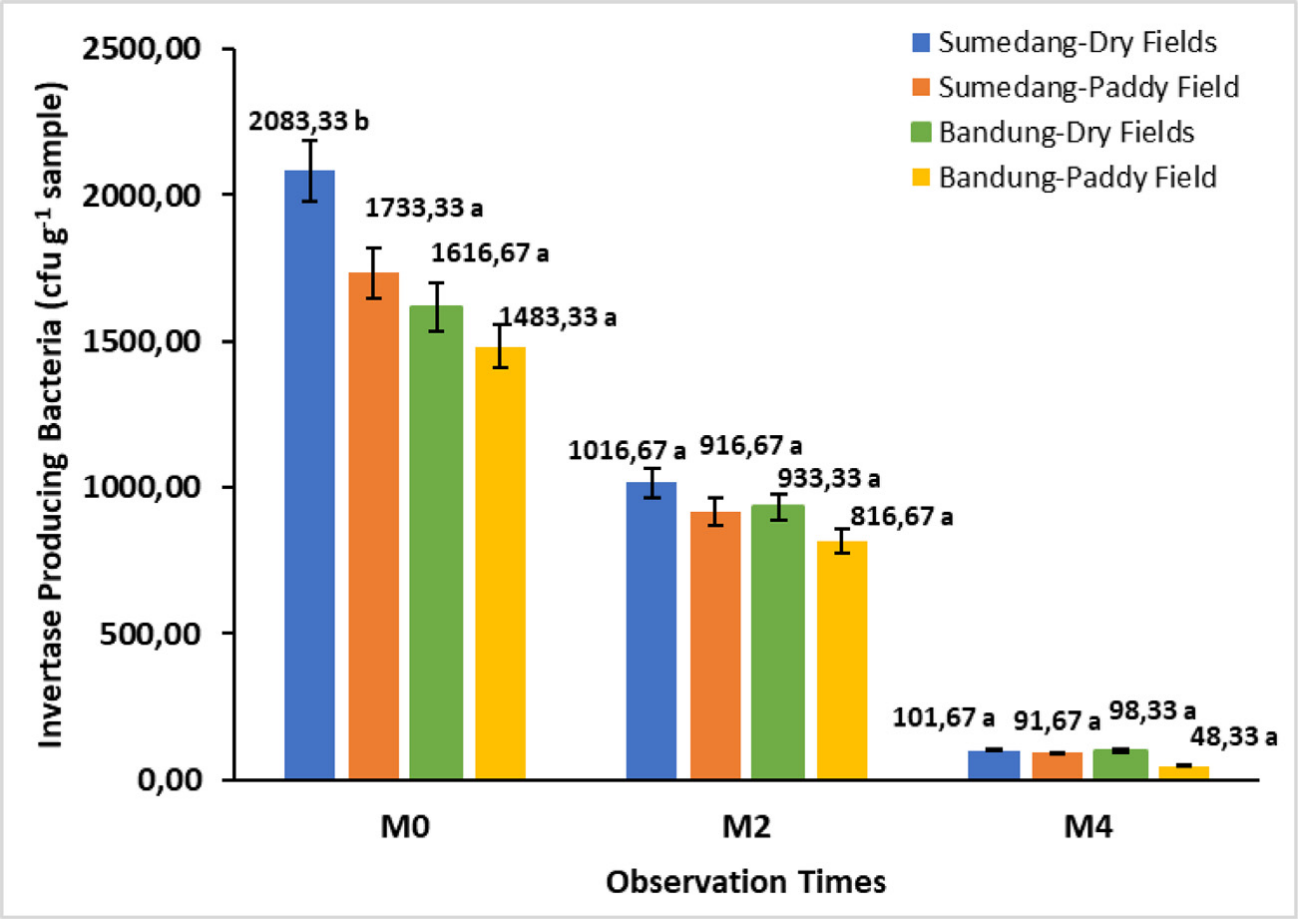
Bar chart of the storage time effect on total invertase-producing bacteria in Cilembu sweet potatoes at various environments. M0= 0 weeks after storage, M2= 2 weeks after storage, M4= 4 weeks after storage. Numbers in the same bar chart followed by the same letter are not significantly different based on the Duncan test at the 5 % level.

The data in Fig. 4 shows the abundance of invertase-producing bacterial populations on sweet potatoes during harvest (M0), two weeks after storage (M2), and four weeks after storage (M4). The population of invertase-producing bacteria decreased during storage, where the decrease was very significant, namely 42.27 % to 51.20 % in M2, and in M4, around 89.46 % to 94.08 %.

**Fig. 5:**
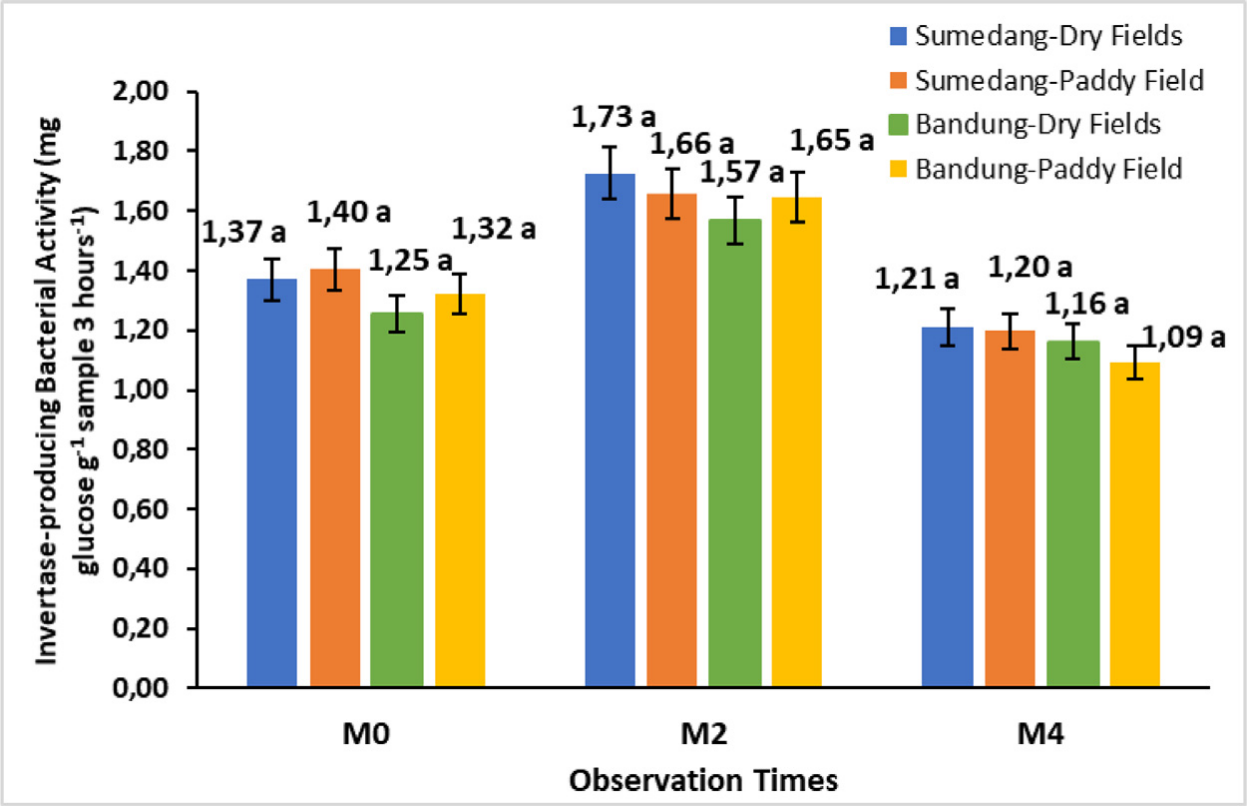
Bar chart of the storage time effect on the activity of invertase-producing bacteria in Cilembu sweet potatoes at various locations. M0= 0 weeks after storage, M2= 2 weeks after storage, M4= 4 weeks after storage. Numbers in the same bar chart followed by the same letter are not significantly different based on the Duncan test at the 5 % level.

The data in Fig. 5 shows invertase activity in sweet potatoes. Invertase activity in M2 increased in all environments, ranging from 18.57 % to 26.28 %. Meanwhile, M4 showed a decrease in invertase activity of around 26.11 % to 33.94 %.

**Fig. 6:**
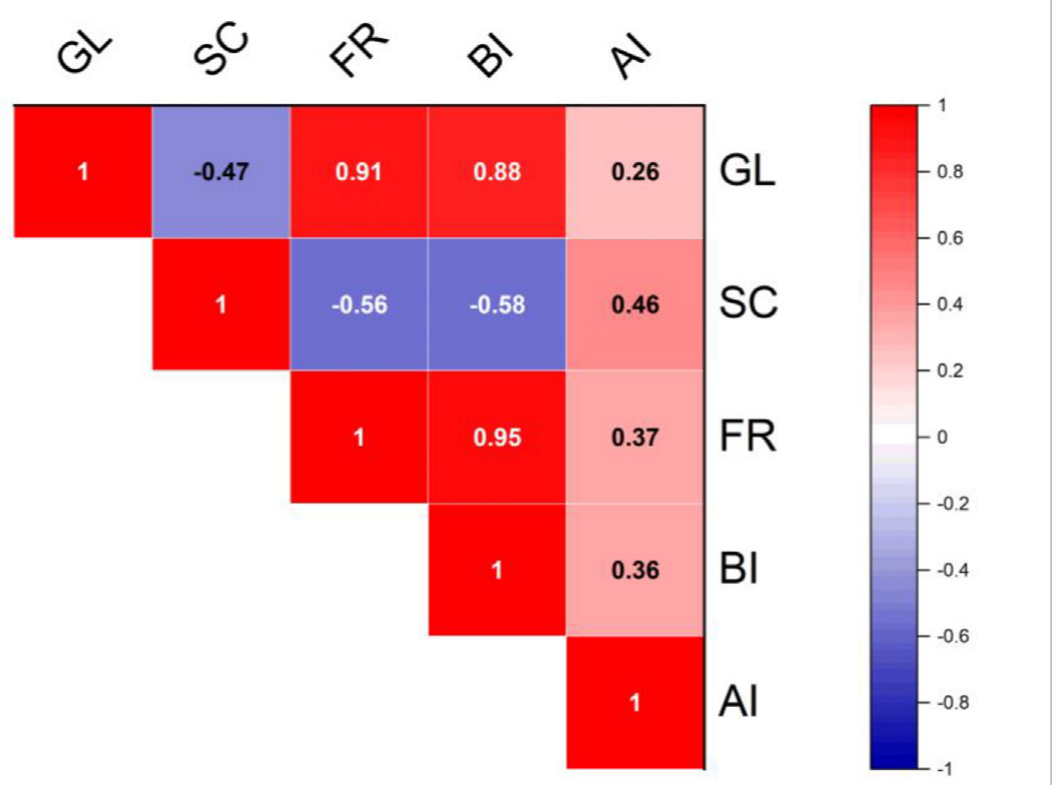
Correlation between sugar content, invertase-producing bacteria, and invertase activity. The dark color indicates a higher correlation. The red color indicates a positive correlation, and the blue color indicates a negative correlation. GL=Glucose, SC=Sucrose, FR=Fructose, BI=Invertase - producing bacteria, AI=Invertase - producing bacteria activity.

The data in Fig. 6 shows the relationship between sugar content and invertase-producing bacteria. Sucrose content had a negative and significant correlation with fructose (−0.56) and invertase-producing bacteria (−0.58). Glucose levels were significantly and positively correlated with fructose levels (0.91) and invertase-producing bacteria (0.88). Fructose levels also significantly and positively correlated with invertase-producing bacteria (0.95).

## 4 Experimental Design, Materials and Methods

### 4.1 Experimental design

This research used a factorial Randomized Completed Block Design, repeated thrice. The first factor is sweet potatoes planted in various locations consisting of Sumedang - paddy fields, Sumedang - dry fields, Bandung - paddy fields, and Bandung - dry fields. The second factor is storage time, which consists of three levels, namely 0 weeks after storage (M0), 2 weeks after storage (M2), and 4 weeks after storage (M4). The additive linear model used is as follows [7]:

Yijk=μ+ai+ßj+(aß)ij+σk+εijk

Keterangan: Yijk: Observation of location treatment at the i^th^ level, storage time at the j^th^ level and k^th^ replication. µ: General average value. αi: environment effect. ßj: storage time effect. (αß)ij: effect of interaction between location and storage time factors. Σk: additive effect of groups. Єijk: random effect of location treatment at the i^th^ level, storage time at the j^th^ level and k^th^ replication.

Based on this model, each observation was hypothesis tested to identify the effect of planting location, storage time, and their interactions on glucose, sucrose, fructose levels, invertase abundance and activity.

## 5 Materials

### 5.1 Sugar analysis

The sugar content observed was determined to be sucrose, glucose, and fructose using the method of [8]. Samples were taken from sweet potatoes stored at M0, M2, and M4, amounting to 1 g. The sample was ground, put into a 25 mL measuring flask, and dissolved in bides water. The mixed solution was shaken and filtered using Whatman filter paper 45. Two (2) mL of the supernatant were put into a vial and then into the High-Performance Liquid Chromatography (HPLC) sample chamber. The external standards used are glucose, fructose, and sucrose. Standard solutions start from 0.25 %, 0.5 %, 1.0 %, and 2.0 %. Fructose retention time is around 8 min, glucose 9 min, and sucrose 10 min.

### 5.2 Enumeration of the invertase-producing bacteria abundance

Abundance enumeration of invertase-producing bacteria was observed by directly observing total microbial counts. Preparation includes taking sweet potato samples at each storage time, namely M0, M2, and M4. Sweet potato samples were cleaned of adhering dirt, washed with running water, and cut into several pieces. The sample was surface sterilized using 70 % alcohol for 1 min, 0.1 % HgCl solution for 1 min, sterile distilled water for 1 min, then ground and taking about 10 g was put into an Erlenmeyer containing 95 mL of physiological solution (0.85 % NaCl) sterile then homogenized using a shaker at a speed of 120 rpm for 15 min. The homogenized sweet potato suspension was diluted up to 10–6. Each dilution level 10–2 to 10–6 was inoculated as much as 0.1 mL in a petri dish containing selective sucrose hydrolysis media, each dilution with two repetitions [9]. The culture is incubated for 24–48 h at room temperature. Observations were made by counting the number of bacterial colonies that changed color to red when sprayed with 0.1 % TTC.

### 5.3 Invertase activity analysis

Invertase activity in sweet potatoes was observed using the method of [10]. One sweet potato was taken from the samples stored at each storage time, namely M0, M2, and M4. Ground and 5 g were taken, then put into a test bottle, and 5 mL of 6 % sucrose substrate was added to the sweet potato sample and 5 mL of distilled water. For control, then shaken briefly and incubated at 50 oC for 3 h. After incubation, 5 mL of distilled water was added to bring the volume to 10 mL, then shaken briefly and filtered to take the filtrate. A total of 1 mL of filtrate was put into a test tube and diluted ten times. 1 mL of the diluted filtrate was put into a test tube, 1 mL of reagent A and 1 mL of reagent B were added, then homogenized using a vortex. The solution was incubated for 15 min in boiling water and then cooled to room temperature. 5 mL of reagent C was added to each test tube and homogenized. The solution was left at room temperature for 60 min, and a color change was observed. Absorbance was measured by a spectrophotometer at a wavelength of 690 nm.

Standard series construction: A total of 0 (no reagent); 0.1; 0.2; 0.3; 0.4; 0.5, and 0.6 mL of the working standard solution were put into 7 test tubes, then distilled water was added to each to a volume of 1 mL and the absorbance was measured with a spectrophotometer at a wavelength of 690 nm.

## 6 Methods

To determine differences in the number and activity of invertase-producing bacteria in various planting environments, they were analyzed using analysis of variance (ANOVA) and followed by a mean value test using Duncan's Multiple Range Test (DMRT) at a confidence level of 95 %. The relationship between bacterial activity and total sugar content was estimated using Pearson correlation analysis following the equation:

$$r=\frac{\left[n\left(\sum xy\right)-\sum x\sum y\right]}{\sqrt{\left[n\left(\sum x^{2}\right)-{\left(\sum x\right)}^{2}\right]\;\left[n\left(\sum y^{2}\right)-{\left(\sum y\right)}^{2}\right]}}$$

Where, x is the independent variable, y is the dependent variable, n is the sample size, and Σ represents a summation of all values.

## Limitations

Not applicable.

## Ethics Statement

The dataset described in this article does not involve any human subjects, animal experiments, or data collected from social media platforms.

## CRediT authorship contribution statement

**Eso Solihin:** Conceptualization, Methodology, Software, Validation, Resources, Writing – review & editing, Project administration, Funding acquisition. **Syaiful Anwar:** Conceptualization, Supervision, Validation, Writing – review & editing. **Dwi Andreas Santosa:** Conceptualization, Supervision, Validation, Writing – review & editing. **Budi Nugroho:** Validation, Writing – review & editing. **Purwono:** Validation, Writing – review & editing. **Rija Sudirja:** Validation, Writing – review & editing, Funding acquisition. **Haris Maulana:** Conceptualization, Methodology, Software, Validation, Resources, Writing – review & editing. **Nadia Nuraniya Kamaluddin:** Validation, Resources, Writing – review & editing. **Agung Karuniawan:** Validation, Resources, Writing – review & editing.

## Data Availability

Invertase-producing bacteria and the sweetness content of sweet potato (Original data) (Mendeley Data).

## References

[1] Maulana H., Solihin E., Trimo L., Hidayat S., Wijaya A.A., Hariadi H., Amien S., Ruswandi D., Karuniawan A. (2023). Genotype-by-environment interactions (GEIs) and evaluate superior sweet potato (Ipomoea batatas [L.] Lam) using combined analysis and GGE biplot. Heliyon.

[2] Solihin M.A., Sitorus S.R.P., Sutandi A. (2018). Widiatmaka, Discriminating land characteristics of yield and total sugar content classes of cilembu sweet potato (Ipomoea batatas L.). Agrivita.

[3] Maulana H., Nafi'ah H.H., Solihin E., Ruswandia D., Arifin M., Amien S., Karuniawan A. (2022). Combined stability analysis to select stable and high yielding sweet potato genotypes in multi-environmental trials in West Java, Indonesia. Agric. Nat. Resour..

[4] Wu X., Wu Z., Ju X., Fan Y., Yang C., Han Y., Chen W., Tang D., Lv Changwen, Cao Q., Wang J., Zhang K. (2023). IbInvInh2, a novel invertase inhibitor in sweet potato, regulates starch content through post-translational regulation of vacuolar invertase IbβFRUCT2. Plant Physiol. Biochem..

[5] Ruan Y.L., Jin Y., Yang Y.J., Li G.J., Boyer J.S. (2010). Sugar input, metabolism, and signaling mediated by invertase: roles in development, yield potential, and response to drought and heat. Mol. Plant.

[6] Minami A., Kang X., Carter C.J. (2021). A cell wall invertase controls nectar volume and sugar composition. Plant J..

[7] Montgomery D.C. (2013).

[8] Zhang Z., Wheatley C.C., Corke H. (2002). Biochemical changes during storage of sweet potato roots differing in dry matter content. Postharvest Biol. Technol..

[9] Reedy P.P., Reddy G., Sulochana M. (2010). Screening of β-fructofuranosidase procedurs with high tranfructosylation activity and its 32 experimental run studies on reaction rate of enzyme. J. Biol. Sci..

[10] Schinner F., von Mersi W. (1990). Xylanase-, CM-cellulase- and invertase activity in soil: an improved method. Soil Biol. Biochem..

